# Pet Rodents and Fatal Lymphocytic Choriomeningitis in Transplant Patients

**DOI:** 10.3201/eid1305.061269

**Published:** 2007-05

**Authors:** Brian R. Amman, Boris I. Pavlin, Cesar G. Albariño, James A. Comer, Bobbie R. Erickson, Jennifer B. Oliver, Tara K. Sealy, Martin J. Vincent, Stuart T. Nichol, Christopher D. Paddock, Abbigail J. Tumpey, Kent D. Wagoner, R. David Glauer, Kathleen A. Smith, Kim A. Winpisinger, Melody S. Parsely, Phil Wyrick, Christopher H. Hannafin, Utpala Bandy, Sherif Zaki, Pierre E. Rollin, Thomas G. Ksiazek

**Affiliations:** * Centers for Disease Control and Prevention, Atlanta, Georgia, USA; † Ohio Department of Agriculture, Reynoldsburg, Ohio, USA; ‡ Ohio Department of Health, Columbus, Ohio, USA; § Arkansas Department of Health and Human Services, Little Rock, Arkansas, USA; ¶ Arkansas Department of Agriculture, Little Rock, Arkansas, USA; # Rhode Island Department of Environmental Management, Providence, Rhode Island, USA; ** Rhode Island Department of Public Health, Providence, Rhode Island, USA; 1 These authors contributed equally to this article.; 2 Current affiliation: Johns Hopkins Bloomberg School of Public Health, Baltimore, Maryland, USA; 3 Current affiliation: Ithaca College, Ithaca, New York, USA

**Keywords:** LCMV, hamster, outbreak, transmission by organ transplantation, zoonoses, Mesocricetus auratus, research

## Abstract

In April 2005, 4 transplant recipients became ill after receiving organs infected with lymphocytic choriomeningitis virus (LCMV); 3 subsequently died. All organs came from a donor who had been exposed to a hamster infected with LCMV. The hamster was traced back through a Rhode Island pet store to a distribution center in Ohio, and more LCMV-infected hamsters were discovered in both. Rodents from the Ohio facility and its parent facility in Arkansas were tested for the same LCMV strain as the 1 involved in the transplant-associated deaths. Phylogenetic analysis of virus sequences linked the rodents from the Ohio facility to the Rhode Island pet store, the index hamster, and the transplant recipients. This report details the animal traceback and the supporting laboratory investigations.

## Abstract 2

A unique strain of this virus was traced back to hamsters from an Ohio rodent distribution facility.

Lymphocytic choriomeningitis virus (LCMV) is a rodentborne arenavirus endemic in house mice (*Mus musculus*) worldwide (*1*–*3*). Lymphocytic choriomeningitis (LCM) in immunocompetent persons usually is a mild, self-limited, viral syndrome or is asymptomatic; aseptic meningitis also can occur, but the infection is rarely fatal (*4*–*6*). In immunocompromised persons, LCM may result in serious systemic infections and death. LCM during pregnancy can cause spontaneous abortion or severe birth defects, including hydrocephalus, chorioretinitis, blindness, or psychomotor retardation (*7*). Congenital LCMV infection is likely greatly underreported as a cause of poor pregnancy outcomes (*7*). Human infection occurs most commonly through exposure (by direct contact or inhalation of infectious aerosol) to secretions or excretions of infected animals (*8*). To our knowledge, person-to-person transmission has not been reported, except for transmission from mother to fetus (*7*) and 1 previous cluster in December 2003 of infection through organ transplantation (*9*,*10*).

In early April 2005, 4 recipients of solid-organ transplants in 3 hospitals in Rhode Island and Massachusetts became gravely ill shortly after transplantation; 3 subsequently died (*10*). All 4 recipients shared a common donor. Tissue and blood samples from the donor and recipients were sent from the Rhode Island Department of Health and the Massachusetts Department of Public Health to the Centers for Disease Control and Prevention (CDC), where LCMV was identified as the etiologic agent (*10*). Viral sequences from the organ recipients were identical to those from a pet hamster acquired by the donor’s household 17 days before organ donation (*10*). Here we report the results of an epidemiologic and environmental investigation to identify the origin of the index hamster and the source of the virus.

## Methods

### Epidemiologic Investigation

Thorough epidemiologic investigations were conducted at the Rhode Island pet store where the index hamster was purchased, the Ohio distribution facility that supplied the pet store, and the primary breeding facility in Arkansas. These investigations focused on interviews; review of invoices, shipping records, and US Department of Agriculture inspection reports; and on-site environmental assessments.

### Rodent Sample Collection

All available rodent species known to be competent hosts for LCMV (capable of becoming chronically infected and shedding virus for up to 9 months) (*6**,**11**,**12*) were collected from the remaining rodent stock at the Rhode Island pet store. These species included Syrian hamsters (*Mesocricetus auratus*), “fancy” mice (*Mus musculus*), and guinea pigs (*Cavia porcellus*). Although they have not been shown to be competent reservoirs for LCMV, “fancy” rats (*Rattus norvegicus*) and gerbils (*Meriones unguiculatus*) also were sampled because of their exposure to the infected rodents. Rodents were sampled and euthanized following approved CDC Animal Care and Use Committee protocols.

With a known population size and a LCMV prevalence estimate, the hypergeometric probability distribution was used to determine the minimum sample size needed to provide a 95% chance of detecting at least 1 LCMV infected rodent at each site. The LCMV prevalence was estimated to be 4.7% in Ohio and 4.3% in Arkansas. The Ohio prevalence was based on 4 infected of 85 tested at the Rhode Island pet store; the revised prevalence for Arkansas was based on 9 of 211 positives after data from the Ohio samples were incorporated.

The population sizes (Table 1) included only dwarf hamsters and did not distinguish between the Chinese and Roborovsky dwarf hamsters (*Cricetulus curtatus* and *Phodopus roborovskii,* respectively). An agreement was reached with the owner in which ≈10% of the total population of 140 Roborovsky’s dwarf hamsters were sampled. In this case, the probability of detecting at least 1 positive rodent was 36.5%.

**Table 1:** Estimated population sizes and samples taken from 2 rodent distribution facilities

Location	Species	Population size	Projected prevalence, %	Sample size	Probability of detecting a positive, %
Ohio	Syrian hamsters	5,000	5.0	116	99.8
Arkansas	Fancy rats	>10,000	3.4	125	98.7
Arkansas	Fancy mice	200	3.4	75	96.5
Arkansas	Gerbils	2,500	3.4	125	98.8
Arkansas	Dwarf hamsters	3,750	3.4	113*	98.1
Arkansas	Roborovsky dwarf hamster	140	3.4	12†	36.5

*Sample size after removal of 12 Roborovsky hamsters from requested sample size. †Sample size represents an agreed-upon portion of the total population.

### Laboratory Investigation

The index hamster, the organ recipients, the animals from the pet store, and rodents from the Ohio and Arkansas facilities were tested for LCMV with a combination of assays that included serology, immunohistochemistry (IHC), reverse transcription–PCR (RT-PCR), TaqMan (Applied Biosystems, Foster City, Ca, USA), and virus isolation. Genetic sequences obtained from the respective samples were used in the phylogenetic analysis to identify the LCMV strain and epidemiologic link leading to transplant-associated deaths.

### Virus Isolation

Virus isolation was conducted by using Vero E-6 cells. For blood or serum, 100 μL of sample was used as inoculum. For tissues, a 10% cell suspension was prepared in a viral support medium (Hank’s balanced salt solution with 5% heat-inactivated fetal bovine serum) and clarified by centrifugation. A100-μL aliquot of the supernatant fluid was used as the inoculum. Flasks were incubated for 1 hour, fed with maintenance medium, and observed for 2 weeks. Cells from flasks were tested for replicating LCMV by immunofluorescent antibody assay (IFA) on 1 of days 4–7 (depending on supplemental information made available through other testing) and again on day 14. If no reactivity was detected by IFA from days 4–7 or on day 14, the flask was considered negative for virus.

### Molecular Detection of LCMV in Rodents

Highly sensitive real-time RT-PCR TaqMan assay was performed as described previously (*10*). RNA isolated from rodent blood, serum specimens, or tissue was subjected to TaqMan real-time assay, and samples with cycle threshold (Ct) values <40 were scored as LCMV-positive. TaqMan-positive specimens were further analyzed by traditional RT-PCR to produce a 232-nt product within the RNA polymerase (L) gene and sequences were obtained by using previously described primers (*10*). The sequences of LCMV from the transplant recipients, index hamster, and rodents from the Rhode Island pet store and Ohio distribution center were then compared with those obtained for other characterized LCMV strains by using GCG Version 11.1.1 (Accelrys, San Diego, CA, USA) and PAUP (Sinauer Associates Inc., Sunderland, MA, USA). Further evidence of a genetic link between LCMV detected in the rodents and the human cases investigated was obtained by analyzing the viral S RNA segment. A 611-nt S segment PCR product was amplified by using 1-step RT-PCR protocols with a generic primer set (*13*) capable of amplifying Old World arenaviruses including LCMV. The 1-step RT-PCR was carried out by using the SuperScript III One-Step RT-PCR System with Platinum Taq High Fidelity as described by the manufacturer (Invitrogen, Carlsbad, CA, USA).

### Serologic and Immunohistochemical Detection of LCMV in Rodents

An ELISA was used to evaluate serum samples collected from rodents for immunoglobulin class G (IgG) antibodies that reacted with LCMV antigens produced in Vero E-6 cells. The assay was run as described in Fischer et al. (*10*), except that a protein A/protein G conjugate (Immunopure, Pierce Biotechnology Inc., Rockford, IL, USA) was used. A subset of the samples was also tested by IFA, using infected Vero E-6 cells. Immunohistochemical tests were carried out on a variety of tissues from the index hamster (blood, adrenal gland, salivary gland, pancreas, liver, spleen, kidney, lung, heart, bone marrow, cerebrum, cerebellum, brain stem, spinal cord) as previously described by Fischer et al. (*10*).

## Results

### Rhode Island Traceback Investigation

Physical inspection of the pet store where the donor’s hamster was purchased produced no evidence of wild rodent infestation. The store maintained live-capture traps in areas likely to harbor rodents (e.g., near feed bags); the trapping log showed no captures in the preceding 3 months.

Although other rodents had been housed in the same area of the pet store with the index hamster, detailed records were not available and these specific rodents were not identified. Invoices dated from February through March 2005 confirmed that all rodents sold at the pet store had come from the Ohio facility.

Biosecurity in the store was limited, with opportunity for interspecific and intraspecific cross-infection, particularly due to a lack of employee hand hygiene between handling of individual rodents. As a precautionary measure, all rodents were quarantined at the store and further sales were prohibited by the Rhode Island Department of Health after LCMV was identified in the organ recipients and the index hamster.

A total of 85 animals (55 hamsters, 8 guinea pigs, 10 mice, 7 gerbils, and 5 rats) were sampled from the remaining quarantined rodent stock at the Rhode Island pet store. Of these, 1 guinea pig and 2 hamsters were found positive for LCMV by several methods (Table 2). LCMV antibodies were detected in 1 hamster by IFA, but not ELISA. LCMV isolates were obtained from either blood or kidney and immunohistochemical stains were positive in at least 1 organ in each of the 3 rodents. All 3 rodents were positive for LCMV RNA with the L-gene–specific TaqMan primer/probe set. The L-gene sequences obtained from these rodents were identical to one another and differed from the index hamster and transplant recipients by only 1 nt (Figure, panel A). Further evidence confirming the presence of viruses of the same genetic lineage in this episode was gathered by RT-PCR amplification of a product from the S segment. The 611-nt S segment sequences of the index hamster and the transplant recipients were 100% identical, thereby reconfirming the previously established genetic link (*10*). In addition, the S segment sequences obtained from the 2 Rhode Island pet store hamsters were identical, and they differed by only 2 nt from the guinea pig sequence (Figure, panel B). These results indicate the same LCMV virus strain was present in the hamsters and guinea pig in the Rhode Island pet store.

**Table 2:** Results of laboratory testing on the index hamster and traceback rodents associated with organ transplantation transmission of LCMV*

Rodent†	IFA	ELISA	IHC	RT-PCR/ TaqMan	Virus isolation	Sequence (L gene)
Index hamster	ND	Neg	Pos	Pos	Pos	232 bp
Pet store hamster 1	Pos	Neg	Pos	Pos	Pos	232 bp
Pet store hamster 2	Neg	Neg	Pos	Pos	Pos	232 bp
Pet store guinea pig 1	ND	Neg	Pos	Pos	Pos	232 bp
Ohio hamster 1	Pos	Neg	Pos	Pos	Pos	232 bp
Ohio Hamster 2	Pos	Neg	Pos	Pos	Pos	NA
Ohio hamster 3	Pos	Neg	ND	Neg	Neg	NA
Ohio hamster 4	Neg	Neg	Pos	Pos	Pos	NA
Ohio hamster 5	Neg	Neg	Neg	ND	Pos	NA

*LCMV, lymphocytic choriomeningitis virus; IFA, immunofluorescent antibody assay; IHC, immunohistochemistry; RT-PCR, reverse transcription–PCR; ND, no data; Neg, negative; Pos, positive; NA, no amplification (could not get traditional PCR primers to amplify, for sequencing). †The table includes only those rodents that tested positive with ≥1 test.

**Figure:**
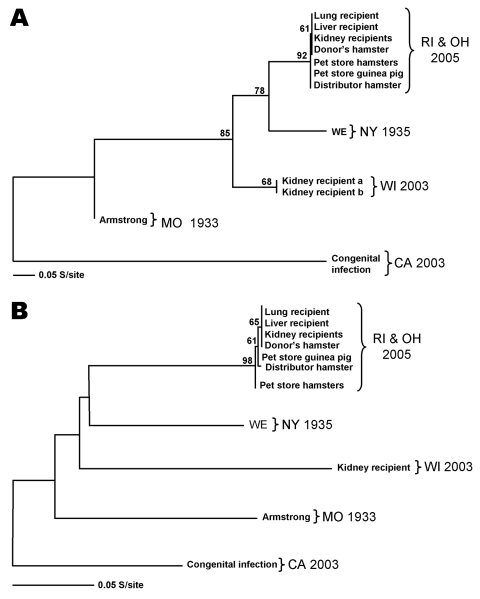
Lymphocytic choriomeningitis (LCM) virus phylogenetic analysis of L- and S-segment sequence differences. A) Maximum likelihood analysis of a 232-nt fragment of the L segment was completed, and bootstrap numbers were generated based on analysis of 500 replicates. The graphic representation was outgrouped to the California (CA) LCM virus sequence. GenBank nos. for the included sequence are as follows: Rhode Island (RI) and Ohio (OH) transplant recipients strain 200501927 (DQ182703), Rhode Island pet store and Ohio distributor rodents strain 200504261 (DQ888889), New York (NY) strain WE (AF004519), Wisconsin (WI) transplant recipients strain 810362 (DQ182706), Missouri (MO) strain Armstrong (J04331), and the CA congenital infection strain 810366 (DQ182707). B) Maximum likelihood analysis of a 611-nt fragment of the S segment NP gene was completed as mentioned above. The GenBank nos. are as follows: RI and OH transplant recipients strain 200501927 (DQ888890), RI pet store guinea pig strain 200502048 (DQ888891),OH distributor hamster strain 200504261 (DQ888893),RI pet store hamsters strain 200501966 (DQ888892), NY strain WE (M22138), WI transplant recipient strain 810362 (DQ182704), MO strain Armstrong (NC_004294), and the CA congenital infection strain 810366 (DQ182705).

### Ohio Traceback Investigation

The Ohio facility served as a distribution/staging area for rodents destined for sale in the northeastern United States. Records indicated that it received most of its hamsters from its parent breeding facility in Arkansas. Both facilities, owned by the person, routinely received shipments of rodents from smaller breeders. The Arkansas facility was the largest distributor in North America, with sales to many states.

Breeding operations at the Ohio facility had been suspended in February 2005 by order of the United States Department of Agriculture (USDA) for multiple violations of the Animal Welfare Act (AWA) (*14*); the suspension included all rodent species other than mice and rats. Interviews and a review of prior USDA facility inspection records indicated ongoing AWA violations and poor biosecurity practices: escaped mice and wild *Mus* spp. that entered the facility were routinely captured and added to captive breeding populations; sick rodents shared common airspace with healthy rodents, attending veterinary services were sporadic, rodents were shipped to Ohio without necessary permits and veterinary inspections, and rodents from different sources shared cages for breeding purposes without adequate quarantine practices.

With the assistance of the Ohio Departments of Agriculture and Health, the Ohio facility was quarantined and inspected on July 18, 2005. During this inspection, several examples of poor biosecurity were found: escaped rodents ran free and entered other holding bins; evidence of a wild rodent infestation was found among the feed sacks; and rodents from disparate sources were housed in adjacent racks, with opportunity for large and small particle cross-contamination.

Shipping records were inadequately maintained, making it difficult to accurately account for the individual shipments of rodents. However, hamsters arriving at the Ohio facility from either Arkansas or other outside breeders were placed in tubs labeled with the location of origin and the date of arrival. According to employees at the facility, these hamsters were not mixed with other shipments.

A sample of 126 rodents (116 hamsters, 9 mice, and 1 guinea pig) was collected. This total comprised the statistically necessary 75 (taken from the general population) plus escaped, sick, and dead rodents. Of the specimens examined, 5 hamsters were positive for LCMV by at least 1 method (Table 2): 3 were positive by IFA, but not by ELISA. Three were positive by IHC; these same 3 were LCMV RNA–positive as evidenced by L-segment–specific TaqMan assay. Virus was isolated from kidney tissues of these 3 hamsters, and sequences were obtained from 1 virus isolate. L-segment sequence comparison of the Ohio specimen found exact identity to the Rhode Island pet store hamster virus (Figure, panel A). Additionally, sequences obtained from the 611-nt S segment of the Ohio hamster differed by only 3 nt from the index hamster sequence, thus showing 99.5% identity (Figure, panel B). These sequences were compared to sequences from other previously identified LCMV strains such as the laboratory strains LCMV-Armstrong and WE, and other isolates from clinical material (Table 3). Differences in L-segment sequences in the viruses from the index hamster and pet store/distribution center rodents were <0.5%, while LCMV-Armstrong and WE differed by 18.1% and 13.4%, respectively. Comparison of the S-segment sequences between the index hamster and pet store/distribution center viruses showed <1% difference. LCMV-Armstrong and WE differed by 14.1% and 14.7%, respectively, from the index hamster virus.

**Table 3:** Comparison of nucleotide identity differences among LCMV strains and isolates*

State/sample	1	2	3	4	5	6	7	8	9	10	11	12	13
Rhode Island													
Lung recipient 1		0.0	0.0	0.0	0.0	0.4	0.4	0.4	0.4	13.4	15.8	18.1	22.9
Liver recipient 2	0.0		0.0	0.0	0.0	0.4	0.4	0.4	0.4	13.4	15.8	18.1	22.9
Kidney recipient A 3	0.0	0.0		0.0	0.0	0.4	0.4	0.4	0.4	13.4	15.8	18.1	22.9
Kidney recipient B 4	0.0	0.0	0.0		0.0	0.4	0.4	0.4	0.4	13.4	15.8	18.1	22.9
Donor's hamster 5	0.0	0.0	0.0	0.0		0.4	0.4	0.4	0.4	13.4	15.8	18.1	22.9
Pet store hamster 1 6	0.3	0.3	0.3	0.3	0.3		0.0	0.0	0.0	12.9	15.3	18.5	23.4
Pet store hamster 2 7	0.3	0.3	0.3	0.3	0.3	0.0		0.0	0.0	12.9	15.3	18.5	23.4
Pet store guinea pig 8	0.2	0.2	0.2	0.2	0.2	0.2	0.2		0.0	12.9	15.3	18.5	23.4
Ohio													
Distributor hamster 9	0.3	0.3	0.3	0.3	0.3	0.3	0.3	0.2		12.9	15.3	18.5	23.4
New York													
WE 10	12.4	12.4	12.4	12.4	12.4	12.1	12.1	12.3	12.3		16.2	18.5	24.1
Wisconsin													
Kidney recipients 11	14.4	14.4	14.4	14.4	14.4	14.4	14.4	14.2	14.4	14.6		19.5	22.5
Missouri													
Armstrong 12	14.1	14.1	14.1	14.1	14.1	13.7	13.7	13.9	14.1	15.2	13.7		21.7
California													
Congenital infection 13	14.7	14.7	14.7	14.7	14.7	14.4	14.4	14.6	14.7	13.3	14.6	14.4	

*LCMV, lymphocytic choriomeningitis virus. The percentage of differences for each pair of sequences was calculated with PAUP as uncorrected distances. Values on the upper diagonal represent differences in the L fragment (232 nt) and values on the lower diagonal represent differences on the S fragment (611 nt)

### Arkansas Traceback Investigation

Several attempts were made to sample the rodents in the Arkansas facility. The initial sample was to include only Syrian hamsters and guinea pigs, but these species were destroyed by the owner when LCMV was found in the Ohio facility. Other remaining rodent species were then sampled as a proxy measure. With the assistance of the Arkansas Department of Health, 450 rodents were sampled, including 125 fancy rats, 125 gerbils, 75 fancy mice, 113 Chinese dwarf hamsters, and 12 Roborovski dwarf hamsters. One fancy rat was IgG positive for LCMV by ELISA. All other test results, including virus isolation, were negative.

## Discussion

This report documents the animal traceback investigation, which linked a major pet rodent distribution operation to the recent outbreak of lymphocytic choriomeningitis in 4 organ transplant recipients in Rhode Island and Massachusetts. This investigation demonstrates the ways in which classic epidemiology, laboratory diagnostics, and molecular biology can complement one another in the investigation of disease clusters. LCMV was not found in the organ donor’s tissues; however, the viral isolate from the pet store hamster was sequenced and matched to the sequences of the isolates from the recipients (*10*). The near-complete sequence match between the virus found in the index hamster and the virus sequenced from the Ohio hamster indicates that the genotypes share a common lineage that is distinct from previously identified strains. It is unlikely that this genotype would be as similar to a genotype found in wild house mice in Rhode Island. Thus, the animal traceback, coupled with the molecular phylogenetic evidence, supports the hypothesis that the index hamster’s infection came from the rodent distribution center in Ohio, rather than from wild *M. musculus* populations around the home of the donor or pet store.

Sequence and phylogenetic data provided strong support for the presence of the same LCMV lineage in hamsters and guinea pigs in the Rhode Island pet store and the Ohio distribution center and established the epidemiologic link of that particular lineage of LCMV to transplant-associated deaths. Comparison of LCMV genotypes obtained from this investigation with previously identified strains LCMV-Armstrong and WE and other isolates from clinical material found considerable differences (Figure, Table 3). While the differences in S-segment sequences between the index hamster and pet store/distribution center viruses were <1% (<3-nt difference), LCMV-Armstrong and WE differed from index hamster sequences by 14.1% (76-nt difference) and 14.7% (86-nt difference), respectively. Similarly, the differences in L-segment sequences in the viruses from the index hamster and pet store/distribution center rodents were <0.5% (3-nt difference), while LCMV-Armstrong and WE differed by 18.1% and 13.4% (41 nt and 30 nt), respectively. These analyses indicate an overall close identity among LCMV strains implicated in this investigation, and wide differences from previously published sequences of strains Armstrong and WE. This molecular evidence corroborates the epidemiologic data implicating LCMV transmission within the commercial pet trade.

After the identification of LCMV in the Ohio facility, all states that had received animals from this facility in the previous 5 months were notified. Many potentially infected animals still remained in stores; their disposition was determined by individual states. Actions taken ranged from sale or adoption with an information leaflet or informed consent to issuing stop sale orders on specific rodent species from the Ohio facility.

Upon notification of the Ohio sample test results, the Ohio Department of Agriculture informed the proprietor of the LCMV contamination in his facility and requested that a written plan for decontaminating the facility and a second plan for keeping the facility LCMV-free in the future be provided. The proprietor responded by depopulating the Ohio facility. The quarantine was lifted after the building was disinfected, but the facility was never reopened.

A direct link between the Arkansas and Ohio facilities was established by the discovery of the marked tubs in Ohio. This enabled the Arkansas Departments of Health and Agriculture (Livestock and Poultry) to issue a Joint Quarantine and Inspection Order. However, several days after depopulating the Ohio facility, the proprietor also destroyed all the Syrian hamsters and guinea pigs at the Arkansas facility. Although efforts were made to sample the remaining rodents at the Arkansas facility in an attempt to pinpoint the source of the virus found in Ohio, the owner refused to allow access to the rodents on several occasions and >4 months elapsed between the initial and the ultimately successful attempts to conduct sampling. Virus isolation on all samples was unsuccessful.

Several factors may have contributed to the lack of virus in the Arkansas breeding facility, including the following: 1) the virus was never there, 2) the destruction of the Syrian hamsters and guinea pigs eliminated the virus from the facility, 3) the elapsed time allowed for the removal of infected animals and subsequent decontamination of the facility, and 4) a complete replacement of the rodent stock was accomplished within the facility. Although the facility was under quarantine for 3 of the 4 months between the first and last attempts at sampling, only sporadic surveillance of the facility was carried out by a governmental authority. Elimination of the virus from the population did not likely occur naturally because LCMV can chronically infect mice and will lead to persistent colonial transmission (*6*,*11*).

Since the time of our investigation, the proprietor’s license has been suspended by the USDA for 5 years for violations of the AWA unrelated to this investigation. The Rhode Island pet retailer who sold the index hamster reportedly ceased business relations with the distributor shortly after the infection was linked to his facilities.

To our knowledge, the Rhode Island outbreak represents the first documented case of fatal LCMV infection involving a pet animal (*10*). In the previous cluster of transplant-related LCMV deaths, no rodent exposure was identified (*9*). Several rodent species that are sold as pets, including hamsters, mice, and guinea pigs, can be incidental hosts of LCMV. These species become infected through contact with infected wild mice, and can pass the infection to humans. Most human LCMV infections are associated with exposure to wild house mice (*6*,*15*); however, several outbreaks have been attributed to laboratory and pet mice and hamsters (*5*,*6*,*16*,*17*). One example is the 1974 outbreak associated with pet hamsters sold by a single distributor. A total of 181 symptomatic cases (46 requiring hospitalization) in persons with hamster contact were identified in 12 states; no deaths occurred (*5*). The outbreak was brought under control by voluntary cessation of sale and destruction of the infected breeding stock.

### Prevention

LCMV surveillance should be a primary concern in the pet rodent industry to avoid entry of this virus into pet trade populations. Because of the ubiquitous distribution of the house mouse, eliminating the natural reservoir of LCMV is not practical. Steps can be taken, however, to exclude wild house mice from homes and businesses. Immunocompromised persons and pregnant women should be advised to avoid close contact all rodents and infested areas. Educational materials should address the risk from exposure to wild mice as well as pet rodents. The virus is not naturally present in pet rodent species and the ease of transmission of the virus from pet rodents to humans may be greater than from wild mice when one considers the nature of the relationship between pet rodents and their owners (i.e., close physical contact). Therefore, every effort should be made to eliminate the virus from pet populations when it is discovered.

LCMV is already actively excluded from laboratory rodent populations, because the infection can be an occupational hazard to laboratory workers who work around infected rodents (*5*), and because inapparent infection can interfere with experimental results in rodent studies (*18*). Economic considerations may prohibit such rigorous biosecurity measures like those used for laboratory animals; however, sentinel surveillance (*19*), adequate veterinary care, exclusion of wild rodents (*20*), and good infection control practices can substantially reduce the opportunity for introduction and spread of LCMV and other pet rodent pathogens in commercial pet populations. Efforts to increase such practices within the pet trade are under way. Adherence to regulations that are already in place for obtaining permits and veterinary inspection of commercial rodent populations can also reduce the likelihood of infection and improve animal welfare.

Further efforts to reduce risk for LCM in pet owners are ongoing. Education is critical in preventing LCM and other pet-related infections. Potential pet owners should choose pets appropriate to their household (*21*). Pregnant women and immunocompromised persons should avoid pet rodents altogether (*22*). Additionally, pet owners should be advised of the possibility of acquiring zoonotic diseases from any pet and of precautions that should be taken to prevent acquiring pet-related infections such as LCM, tularemia (*23*), salmonellosis (*24*), and others. Persons can reduce risk for infection from pet rodents by being attentive to proper hand hygiene and environmental cleaning. Additional information on LCMV is available from the CDC website (*25*).
